# The impact of social protection interventions on treatment and socioeconomic outcomes of people with tuberculosis and their households: Protocol for a systematic review and meta-analysis

**DOI:** 10.12688/wellcomeopenres.18807.1

**Published:** 2023-04-18

**Authors:** Mollie Hudson, Heather Todd, Talemwa Nalugwa, Delia Boccia, Tom Wingfield, Priya B. Shete

**Affiliations:** 1 School of Nursing, University of California, San Francisco, San Francisco, USA; 2 Departments of Clinical Sciences and International Public Health, Liverpool School of Tropical Medicine, Liverpool, UK; 3 School of Medicine, University of Liverpool, Liverpool, UK; 4 Uganda Tuberculosis Implementation Research Consortium, Kampala, Uganda; 5 Department of Infectious Disease Epidemiology, London School of Hygiene and Tropical Medicine, London, UK; 6 World Health Organization Collaborating Centre on Tuberculosis and Social Medicine, Department of Global Public Health, Karolinska Institutet, Stockholm, Sweden; 7 Tropical and Infectious Diseases Unit, Liverpool University Hospitals NHS Foundation Trust, Liverpool, UK; 8 Division of Pulmonary and Critical Care Medicine, University of California, San Francisco, San Francisco, USA; 9 Center for Tuberculosis, University of California, San Francisco, San Francisco, USA

**Keywords:** Tuberculosis, social protection strategies, social protection interventions, socioeconomic support, tuberculosis treatment success, catastrophic costs

## Abstract

**Background: **Tuberculosis (TB) is a leading cause of death due to infectious disease worldwide. People with TB and their households often suffer social and economic losses due to the cost of tuberculosis care. The World Health Organization 2015 End TB strategy called for socioeconomic support through social protection interventions. Social protection has the potential to enable people with TB and their households to break the cycle of TB and poverty, thereby improving both treatment and socioeconomic outcomes. This study aims to evaluate whether people with TB who are recipients of social protection interventions have better treatment and socioeconomic outcomes than those who are not recipients of social protection interventions.

**Methods: **We will systematically review literature published in English between 2012 and 2021 from PubMed, Embase, and Web of Science, and grey literature from Google Scholar and selected, relevant databases. We will include studies that describe a social protection intervention (as defined by the World Bank) and report on TB treatment outcomes and/or socioeconomic outcomes. We will only include studies pertaining to populations in low-and-middle-income countries and/or countries with high TB burden. We will follow the Preferred Reporting Items for Systematic Reviews and Meta-Analyses (PRISMA) guidelines. Study quality will be assessed using the Cochrane Risk of Bias for randomized controlled trials and the Newcastle Ottawa Scale for non-randomised controlled studies. If sufficient quantitative data are available, we will perform a meta-analysis of aggregated outcomes. Lastly, we will use the Grading Recommendations Assessment, Development, and Evaluation to describe the overall quality of evidence.

**Ethics and dissemination:** Ethical approval is not required for this systematic review, as all data extraction and analysis will be conducted on published documents. We will disseminate this protocol through conference presentations. The systematic review has been registered prospectively in the PROSPERO database (registration number CRD42022382181).

## Introduction

Tuberculosis (TB) is one of the leading causes of infectious disease deaths worldwide ^1^. Despite effective and widely available treatment, in 2020, 9.9 million people were infected with tuberculosis and over one million people died from TB ^1^. Thirty countries, most of which are low or low-middle income, are formally designated by World Health Organization (WHO) as “high TB burden countries” ^1^. High TB burden countries account for an estimated 90% of all global TB incident cases ^1^. This is not surprising as TB has long been recognized as a disease of poverty.

The relationship between TB and poverty is cyclical; impoverished individuals tend to have multiple risk factors that make them more susceptible to TB (e.g. crowded living conditions, poor access to care, malnutrition), and being ill with TB often yields both direct (e.g. cost of treatment, travel, and food or nutritional support) and indirect (e.g. inability to work for several months or job loss) negative economic effects ^2, 3^. In combination with HIV prevalence, the relationship between TB and poverty helps account for persistently high death rates from TB in low- and middle-income countries ^1^. Despite effective and affordable treatment options, people with TB often face numerous barriers to care. Such barriers include, but are not limited to, food insecurity, stigma and a lack of psychosocial support, high heath care cost, and geography (e.g. long distance to a health center) ^4, 5^. Further, both the direct and indirect costs of TB disease can lead to catastrophic costs (total TB-related costs >20% of a TB-affected household’s pre-TB annual income ^6^) and dissaving (such as taking out loans, using savings, selling assets). This propagates the cycle of TB and poverty and, indeed, can compound the impoverishment of TB-affected households ^3^.

To mitigate catastrophic costs and improve TB treatment outcomes (such as treatment success or cure), the WHO 2015 End TB Strategy recommends social protection for TB-affected households ^7– 9^. Social protection interventions are broadly defined by the World Bank as systems that “help the poor and vulnerable cope with crisis and shocks, invest in the health and education of their children, and protect the aging population.” ^10^ Such interventions include, but are not limited to, cash transfers, job training interventions, and nutrition support. In sum, these interventions help reduce barriers to care for many diseases, including TB.

Several studies, including systematic reviews, have found that social protection interventions can improve TB treatment outcomes (e.g. increased treatment success, decreased mortality) ^11^. However, there are several limitations to the synthesized evidence to date. The scope of existing review articles is either: too narrow, focusing on extremely focused interventions such as cash transfers ^12^, medication adherence interventions ^13^, and economic incentives and enablers ^14, 15^; or too wide, reporting on extremely wide-ranging interventions with diverse mechanisms of action and outcomes, including psycho-emotional components ^16^, which are associated with significant heterogeneity and are therefore challenging to interpret. Additionally, to date, no review has measured the impact of social protection on incurrence of catastrophic costs or other socioeconomic outcomes of interest. Lastly, since 2015, multiple large-scale trials of social protection for TB-affected individuals and households have been undertaken and the evidence base has expanded significantly. More up-to-date evidence could provide meaningful information about the potential impact of socioeconomic interventions on TB treatment and socioeconomic outcomes, as well as information relating to the operational and logistical elements of social protection interventions.

### Objectives

This systematic review and meta-analysis will aim to answer the following questions:

    1. Do people with TB who have enrolled in and/or been recipients of at least one social protection intervention demonstrate an improvement in TB treatment success (completion of treatment of cure) when compared to people with TB who have not enrolled in and/or been recipients of social protection interventions?

    2. Do people with TB who have enrolled in and/or been recipients of at least one social protection intervention have better socioeconomic outcomes, including lower rates of catastrophic costs, when compared to people with TB who have not enrolled in and/or been recipients of social protection interventions?

### Strengths and limitations of this study

***Strengths***

- Screening of potentially eligible studies will be conducted by two researchers independently
- Data extraction and quality assessment will be conducted by three researchers independently
- Standardized definitions of interventions and outcome measures will be described.

***Limitations***

- Heterogeneity between studies describing different types of social protection interventions may make it difficult to pool outcomes and conduct meta-analyses
- Only including studies published in English may limit our findings

## Methods

### Study design

This systematic review protocol will be guided by the Preferred Reporting Items for Systematic Reviews and Meta-analysis protocol (PRISMA-P) checklist (see *Reporting guidelines* ^17^). Briefly, these steps will include generating a search strategy, screening abstracts and articles by the specified inclusion and exclusion criteria, and extracting and synthesizing data from included articles.

### Eligibility criteria

***Study characteristics***

We will include randomized controlled trials, cross-sectional, cohort, cost-effectiveness analyses, modeling, ecological, or quasi-experimental studies in this systematic review. We will only include studies in which the main independent variable is enrollment in a social protection program and/or receipt of at least one social protection intervention, and the main dependent variable is at least one outcome related to TB treatment outcomes and/or socioeconomic outcomes. Outcomes have been determined by selecting standardised TB treatment outcomes used by WHO and well-recognised socioeconomic outcomes used by WHO, the World Bank and United Nations. Outcome measures are described in detail in Table 1.

**Table 1.** Outcomes by PICOT (patient, intervention, comparison, outcome and time). TB, tuberculosis.

**Outcomes for PICOT #1:** Primary and secondary outcomes related to TB treatment	○ *Primary TB treatment outcome:* ■ TB treatment success ■ Death ○ *Secondary TB treatment outcomes:* ■ Cure ■ Treatment completion ■ Adverse TB treatment outcomes: ● Loss to follow up ● Relapse ● Treatment failure ○ While this terminology as no longer used, it is likely that studies will have used this terminology. ● No evaluation
**Outcomes for PICOT #2:** Primary and secondary outcomes related to socioeconomic outcomes.	○ Catastrophic costs ■ Catastrophic costs (total costs of entire TB illness >20% of the same household’s annual pre-TB income) ■ Costs ● Direct medical ● Direct non-medical ● Indirect (lost income, time, and productivity) ○ Of note, these metrics may be calculated different based on the study approach, which will have to be taken into account when analysing our findings. ○ Dissaving ■ Dissaving ● If the patient/household took out a formal or informal loan ● If the patient/household sold an asset or item ● If the patient/household used savings ● If the patient/household took a child out of school ● Reduced household food consumption ○ Percent poor ^18^ based on multidimensional poverty index scores ■ Percent poorer than median poverty score (person with TB and/or TB affected household) ● Experiencing extreme poverty ● Below specified higher poverty lines (USD $3.20 or $5.50 (TB affected household) ● % below SPL (TB affected household) ■ Person with TB and/or TB affected household’s perception of poverty and the impact of TB on their poverty ● For example, if a study used the WHO TB Patient Cost Survey, which asks questions about how TB illness has affected individual and/or household level poverty

***Types of participants***

This systematic review will include people with pulmonary and extra pulmonary TB, people with drug-sensitive (DS-TB) and drug-resistant (DR-TB), people with HIV-TB co-infection, and their TB-affected households, with results disaggregated accordingly.

***Setting and time frame***

The systematic review will only include studies pertaining to low-to-middle-income countries (LMICs) and/or high burden TB countries, and will include studies published between 2012 and 2021 The time frame for eligibility (2012–2022) was chosen based on the “World Bank’s Social Protection and Labour Strategy 2012–2022,” ^19^ in which the World Bank focused their initiatives on reducing socioeconomic risk and strengthening social protection interventions. Additionally, *TB specific* interventions are defined as social protection interventions that target people with TB or TB affected households, with the intention of improving outcomes related to TB. *TB sensitive* interventions are designed to reach, individuals who are at risk of TB infection or disease, but is not limited to those with disease and often include targeting or enrolment based other non-TB characteristics ^20^. Given the study’s overall goal of supporting programmatic implementation of social protection interventions, we will focus on *TB specific* social protection programs. All included search terms are listed in Table 2.

**Table 2.** Search strategy keywords.

	Generic keywords	Other keywords
**1**	Tuberculosis (“TB treatment terms)	TB, Mycobacterium tuberculosis, pulmonary TB, TB-affected, TB-infected, TB patients, drug-resistant TB, TB individuals/households, TB prevalent, TB cases Pulmonary TB, PTB
**2**	Social protection (“intervention terms”)	Social safety net Socioeconomic support Social support Economic support Financial support Cash transfers; food-based programs, supplementary feeding programmes, food stamps, vouchers, and coupons; in-kind transfers such as school supplies and uniforms; conditional cash transfers; price subsidies for food, electricity, or public transport; public works programmes; and fee waivers and exemptions for health care, schooling, and utilities, welfare Food baskets, food rations Protections against shocks Social risk management Transportation Government financing Reimbursement Low and middle income, LMIC Support groups, education, community support
**3**	Support (“intervention terms”)	Intervention, incentive, program, scheme, policy, assistance, livelihood support, enabler
**4**	Impact (“outcome terms”)	Affect, effect, association, associated, consequence
**5**	Treatment (“outcome terms”)	Outcome, success, rates, unsuccessful, uptake, enrolment, adherence, cured, completed, treated, follow-up, loss to follow-up, relapse, recurrence, adverse outcome, diagnostic pathways, TB testing, quality of life, default, care cascade
**6**	Socioeconomic (“outcome terms”)	Outcome, financial burden, economic burden, economic consequences, social consequences, socioeconomic consequences, social impact, socioeconomic impact, costs, expenditure, expenses, spending, catastrophic expenditure, catastrophic costs, impoverishment, coping strategies, poverty, food security, loans, sold assets, dissaving, deprivation, defray, mitigate

***Report characteristics***

Only peer reviewed studies and reports that have been published in English will be included.

***Information sources***

We will search the following three electronic databases: PubMed (includes MEDLINE), Embase, and Web of Science for relevant publications. If an eligible article is missing individual level data and it is not possible to perform analysis on clusters, or if analysis is limited because data is presented in an aggregate form, we will contact the study authors to obtain the data required.

We will use Google Scholar Advanced to search selected, relevant databases with a limited number of search terms (e.g. the WHO or World Bank databases) to identify relevant articles in the grey literature.

***Search strategy***

Table 3 shows examples of search strategies (PubMed and Web of Science). If more than six months have lapsed between the date of the last search and the date of journal submission, we will repeat our search. From the articles deemed eligible for inclusion, two researchers (HT and MH) will also employ snowballing to assess for additional potentially relevant articles. MH will search for potentially relevant articles in the original list of articles found in the initial search to determine if the articles were missed by the search query or if they were screened out by one of the eight screeners (see Table 4 for information on the study team).

**Table 3.** Search strategies.

3A. PubMed search strategy		
#	Searches	Results
1	(Tuberculosis[Title/Abstract] OR TB[Title/Abstract] OR "Mycobacterium tuberculosis" [Title/Abstract] OR "Pulmonary TB" [Title/Abstract] OR "TB affected" [Title/Abstract] OR "TB infected" [Title/Abstract] OR "TB patients" [Title/ Abstract] OR "Drug-resistant TB" [Title/Abstract] OR "TB individuals" [Title/Abstract] OR "TB affected households" [Title/Abstract] OR "TB prevalent" [Title/Abstract] OR "Pulmonary tuberculosis" [Title/Abstract] OR "Pulmonary TB" [Title/Abstract] OR PTB[Title/Abstract]) AND (Social protection[Title/Abstract] OR "Social safety net" [Title/Abstract] OR "Socioeconomic support" [Title/Abstract] OR "Social support" [Title/Abstract] OR "Economic support" [Title/ Abstract] OR "Financial support" [Title/Abstract]) AND ("2012"[Date - Publication] : "2021"[Date - Publication])	308
2	(All TB treatment terms by title and abstract with OR as the Boolean operator) AND (All intervention terms by title and abstract with OR as the Boolean operator) AND ("2012"[Date - Publication] : "2021"[Date - Publication]) (i.e. #1 + additional terms)	17,461
3	(Tuberculosis[MeSH] + all TB treatment terms by title and abstract with OR as the Boolean operator) AND (All intervention terms by title and abstract with OR as the Boolean operator) AND ("2012"[Date - Publication] : "2021"[Date - Publication]) (i.e. #2+ MeSH terms)	17,732
4	("2012"[Date - Publication] : "2021"[Date - Publication]) AND (Tuberculosis[MeSH] OR all TB treatment terms by title and abstract) AND (all intervention terms by title and abstract) AND (all outcome terms by title and abstract) (i.e. #3 + outcome terms).	17,732
3B. Web of Science search strategy		
#	Searches	Results
1	TS=(TB terms with OR as the Boolean operator) AND TS=(all intervention terms with OR as the Boolean operator) AND TS=(all outcome terms with OR as the Boolean operator)	28,985
2	TI=(TB terms with OR as the Boolean operator) AND TS=(all intervention terms with OR as the Boolean operator) AND TS=(all outcome terms with OR as the Boolean operator)	14,687
3	TI=(TB terms with OR as the Boolean operator) AND TI=(all intervention terms with OR as the Boolean operator) AND TI=(all outcome terms with OR as the Boolean operator)	1412
4	TI=(TB terms with OR as the Boolean operator) AND TI=(all intervention terms with OR as the Boolean operator) AND TS=(all outcome terms with OR as the Boolean operator)	3568

**Table 4.** Study team.

Name	Initials	Role
Mollie Hudson	MH	Conceptualization, title and abstract screening, full text review, data extraction, meta-analysis, writing
Heather Todd	HT	Conceptualization, title and abstract screening, full text review, data extraction, meta-analysis, writing
Delia Boccia	DB	Conceptualization
Joseph Kazibwe	JK	Title & abstract screening
Talemwa Nalugwa	TN	Conceptualization
Joseph Pearman	JP	Title & abstract screening
Shreya Puntambekar	SP	Full text review, data extraction
Ann Schraufnagel	AS	Data extraction, risk of bias assessments, meta-analysis
Priya B. Shete	PBS	Conceptualization, full text review, data extraction, meta-analysis, writing
Kristina Skender	KS	Title & abstract screening
Phuong Tran	PT	Title & abstract screening
Tom Wingfield	TW	Conceptualization, full text review, data extraction, meta-analysis, writing

### Study records

***Selection process***

A total of eight reviewers will screen titles and abstracts independently and select articles for full text review. The lead reviewer will hold an initial training session, as well as weekly meetings to address questions as the review progresses. If questions arise about whether to include an article based on title and abstract, this will be discussed as a team with HT and MH making the final decisions. Specifically, two authors (HT and MH) will independently review all titles/abstracts selected for full text review and will come to a consensus with a third reviewer (TN) if MH and HT are not in agreement. Upon reading the full text articles (to be done independently by MH and HT), if there is a disagreement between the two authors about whether a paper should be included in the systematic reviews, authors will discuss with the core group of investigators (PBS, TN, TW) to reach an agreement. We anticipate that MH and HT will be the primary researchers designated to select which articles, and which data (pertaining to outcome measures) can be included in the meta-analysis and will discuss with the core group of investigators should questions arise.

***Data management***

All articles will be imported into Covidence ^21^, where they will be screened for duplicates. All authors will use Covidence ^21^ to conduct title and abstract screening, full text screening, manage records, store data, and detail resolution of disagreement (described further below) throughout the review. Zotero, a free open source software, can be used for similar functions and may be used to replicate this study.

### Data items

Based on our PICOT (patient, intervention, comparison, outcome and time) statements, researchers (AS, HT, MH, SP) will obtain, and document, the following information regarding each eligible study: year, authors, study location, GDP/income classification (at the time of study), whether or not the country is defined as a high TB burden country (or was defined as such at the time of the study), population (adult, pediatric, TB affected household, or unknown), nature of social protection intervention (e.g. food assistance, cash transfer, etc.), and outcomes measured (e.g. TB treatment success, incurrence of catastrophic costs, etc.). Two researchers (AS and MH) will be responsible for extracting quantitative data from reports and conducting risk of bias assessments. Findings will be organized into an Excel table. Study members will independently extract data and will meet to discuss findings and resolve discrepancies.

### Risk of bias in individual studies

We will use the Cochrane Risk of Bias (RoB) tool ^22^ for RCTs, the Newcastle Ottawa Scale (NOS) ^23^ for all other studies that quantitatively report on outcomes, and the Critical Appraisal Skills Program (CASP) ^24^ to assess risk of bias for qualitative studies. Risk of bias will be appraised by two researchers independently (AS and MH) before meeting to resolve discrepancies.

### Data synthesis

If sufficient data is available, we will group outcomes for meta-analyses. Specifically, we anticipate that most studies will report on TB treatment success (defined as cure or treatment completion). We will group studies that report on TB treatment success, cure, or treatment completion as all outcomes meet the criteria for the standardized WHO definition of treatment success. All estimates of effect for dichotomous outcomes (e.g. “achieved treatment success” versus “did not achieve treatment success”) will be reported as risk ratios with a 95% confidence interval. Similarly, we will group studies that report on similar socioeconomic outcomes (e.g. catastrophic costs) and will calculate risk ratios with 95% confidence interval. This information will be considered when deciding which results to pool and how to justify findings/recommendations that may emerge from this systematic review.

The study proposes using a random effects model ^25^ to account for heterogeneity between studies. Results will be presented as forest plots for each strata of interest decision to pool results will be made if there is not either significant statistical or other type of heterogeneity between studies (I ^2^). Investigators will determine a value of I ^2^ above which we will not pool results, as I ^2^ is indicative of how precise the resulting pool is, which will be decided through an iterative process. We also aim to conduct a meta-regression. We anticipate that explanatory variables that may affect the intervention effect will include, but not be limited to, pulmonary versus extrapulmonary TB, DR-TB versus DS-TB, HIV status and type of setting (rural versus urban). From the meta-regression analysis, we will use the regression coefficient to test the relationship between the intervention effect and the explanatory variable ^26^. This will support the team to apply meaning to the results and reflect on the overall findings in the context of successes and challenges of intervention implementation. We anticipate this qualitative analysis will add value of our study findings to policy- and decision-makers and implementers of social protection for TB-affected households. Following data extraction and synthesis, we will use the Grading Recommendations Assessment, Development, and Evaluation (GRADE) to describe the overall quality of evidence. Researchers will prepare a final manuscript using the Preferred Reporting Items for Systematic Reviews and Meta-Analysis (PRISMA) guidelines.

### Patient and public involvement

Patients and members of the public will not be involved in the design, conduct, reporting, or dissemination of the research.

## Discussion

With an extensive list of search terms and queries comprised from expert input, this systematic review aims to yield important information pertaining to the impact of social protection interventions on TB treatment and socioeconomic outcomes in high TB burden, low-middle-income countries. Anticipated findings will build on previously conducted systematic reviews ^27^ and could provide essential, more definitive information about the potential impact of social protection on TB treatment and socioeconomic outcomes. Further, researchers expect that this review will provide key information about the operational and logistical elements of social protection interventions. As noted, this operational guidance would be vital for policy makers and National TB Programs to make informed decisions about which social protection interventions to invest in, implement, and scale-up. Additionally, an anticipated secondary deliverable of the study is a strengthening of the use of the established definition of social protection versus related interventions such as incentives and enablers. This is important because the definitions of incentives and enablers remain poorly and inconsistently defined across governing bodies, multinational organizations, and peer-reviewed publications.

Given that providing people with TB and their households with social protection interventions is part of WHO’s 2015 End TB Strategy, we believe that our findings will be of significant value globally, especially in LMICs and/or high TB burden settings. In addition to evaluating the impact of social protection interventions on TB treatment outcomes, our systematic review and meta-analysis will provide meaningful information regarding catastrophic costs, WHO’s End TB Strategy global indicator of socioeconomic impact. Further, study results could help guide policies regarding social protection interventions for people with TB and TB-affected households. Specifically, we aim to incorporate our findings into the next revision of the WHO TB care and treatment guidelines, and as a case study for the next iteration of the WHO TB Patient Cost Survey Handbook ^28^. We believe that the inclusion of such findings could contribute to efforts to interrupt the cycle of TB and poverty, and subsequently, the effort to reduce the global burden of TB disease.

### Registration

The systematic review has been registered prospectively in the PROSPERO database (registration number CRD42022382181).

### Study status

The study has been registered, the systematic review team has been identified and requisite software and infrastructure established, and abstract/manuscript identification has begun.

## Data Availability

No underlying data are associated with this article. Zenodo: PRISMA-P checklist for ‘The impact of social protection interventions on treatment and socioeconomic outcomes of people with tuberculosis and their households: Protocol for a systematic review and meta-analysis’. https://doi.org/10.5281/zenodo.7702970 ^17^. Data are available under the terms of the Creative Commons Attribution 4.0 International license (CC-BY 4.0).
