# Supplementary material for: The impact of social protection interventions on treatment and socioeconomic outcomes of people with tuberculosis and their households: Protocol for a systematic review and meta-analysis
Source: Wellcome Open Res. 2023 Apr 18;8:175. [Version 1] doi: 10.12688/wellcomeopenres.18807.1 (PMC10511851; doi:10.12688/wellcomeopenres.18807.1)
Supplement: Peer review history

# Reviewer report: Reviewer response for version 1

**DOI:** 10.21956/wellcomeopenres.20853.r63774

**Published:** 2023-09-20

**Authors:** Malaisamy Muniyandi

**Affiliations:** 1 ICMR-National Institute for Research in Tuberculosis,, Chennai,, India

TB is a complex socio economic problem that impedes human development and traps the poorest and most marginalized in a vicious circle of disease and poverty. TB has historically been associated with high levels of poverty, as TB has traditionally been a disease of the poor. The economic impact of TB comes from the size of the problem and from the fact that in developing countries the majority of those affected are in the economically active segment of the population. The theme for the World Tuberculosis Day 2002 is "Stop TB, fight poverty". The theme suggests that tackling TB, one of the several illnesses that affect the poor, is one way of achieving greater global prosperity.

 Though free diagnosis and treatment for TB are available, still there are significant out-of-pocket expenditure incurred by patients and their families. This results in adverse treatment outcomes and has a bidirectional relationship with poverty. This is further disturbed by the financial shocks and this makes the households poorer. In remedy to this, World Health Organisation *End TB Strategy* targets for no TB affected households should face catastrophic cost (>20% of household income) associated with TB. This warrants implementing inventions to provide financial protection by reducing the catastrophic costs.

 Hudson and colleagues, proposing to synthesize the evidence on the impact of social protection interventions on treatment and socioeconomic outcomes of people with TB and their households by a systematic review and meta-analysis. This is a very important topic to study the impact of different strategies to support TB patients from socio-economic aspects. Hudson and team have written the proposal well and discussed the study plan extensively. The proposal covered background of the study, detailed methodology and expected outcomes. The proposing to follow the standard methodology used for systematic review and meta-analysis. The authors can also include the following interventions in addition to mentioned intervention in the proposal: (1) active case finding is one intervention that could result in reduced delays prior to diagnosis, prevent disease transmission in the community and reduce direct and indirect costs for patients and households. 2) To develop public health system or social insurance system that covers the out-of-pocket expenditure to TB patients and their families. (3) Introducing new digital technological interventions and novel approaches such as 99DOTS (a low cost solution for TB medicine adherence), video supported home based TB care, video observed therapy, eHealth portal could also reduce catastrophic costs to the TB patients.   

 This current protocol has clearly mentioned the steps in doing a standard systematic review and meta-analysis by following the PRISMA guidelines. It has also included the steps, which includes generating search strategy, screening abstracts and articles by their specified inclusion and exclusion criteria, which allows the readers to know the potential limitations and future scope of the study. It has clearly explained the PICOT statements and how the data items from the studies will be collected based on the PICOT. It is proposed that the heterogeneity of the study will be assessed using random effects model. Investigators have planned to give a threshold value of I ^2 ^above which the results will not be pooled considering I ^2^ as the indicator of the precision for resulting pool using iteration method. This is highly commendable since the heterogeneity is a factor that highly influences the quality of any meta-analysis if we consider.

 The authors provided a detailed information on how they will synthesize and analyze the data in their study. They aim to do meta-regression wherein they check whether the explanatory variable may affect the intervention effect. This finding will greatly help in visualizing the results and helps us to understand the successes and challenges involved in the intervention implementation. The authors have also mentioned GRADE to describe the quality of evidence, which further adds the quality of the assessment.

 In conclusion, the results of this will serve as a strong scientific evidence for policy makers, stakeholders and TB programme to make decisions about the interventions that can be implemented for the social protection of the people with TB and their households. The results of this study will be useful for strengthening TB programme to achieve the progress towards End TB targets.

Is the study design appropriate for the research question?

Yes

Is the rationale for, and objectives of, the study clearly described?

Yes

Are sufficient details of the methods provided to allow replication by others?

Yes

Are the datasets clearly presented in a useable and accessible format?

Not applicable

Reviewer Expertise:

As a scientist and synergizing more than two decades of health economic research expertise and leadership, I am heading the Department of Health Economics at the ICMR-National Institute for Research in Tuberculosis in Chennai. I have involved in diverse socio-economic, behavioural and epidemiological research and intervention projects in the context of improving population health. I have contributed as a principal investigator and co-investigator in various research projects. I am also responsible for Regional Resource Centre for Health Technology Assessment in India (HTAIn) at ICMR-NIRT, Chennai. I have made significant scientific outputs under my working institutes in terms of documentation and dissemination of research findings. I have a total of 120 research papers published in reputed indexed peer reviewed journals with high impact factors.

I confirm that I have read this submission and believe that I have an appropriate level of expertise to confirm that it is of an acceptable scientific standard.

# Reviewer report: Reviewer response for version 1

**DOI:** 10.21956/wellcomeopenres.20853.r56410

**Published:** 2023-09-08

**Authors:** Bharatkumar Hargovandas Patel

**Affiliations:** 1 Professor and Head, Community Medicine Department, Government Medical College, Bhavnagar, Gujarat, India

At First, I congratulate the authors for publishing the protocol on “The impact of social protection interventions on treatment and socioeconomic outcomes of people with tuberculosis and their households: Protocol for a systematic review and meta-analysis”. I appreciate the hard work done by the authors.

 The review of this protocol was done primarily in line with PRISMA-P checklist and associated article ‘Preferred reporting items for systematic review and meta-analysis protocols (PRISMA-P) 2015: elaboration and explanation’.

 Different sections of this protocol like rationale, objectives, eligibility criteria, information sources, search strategy, study records, risk of bias in individual studies, data synthesis, quality assessment etc. are well described with adequate details.

 However, there are few minor comments for the authors, which will help them in improving the quality of this protocol.

1. **Outcomes by PICOT (Table 1)**
**a**. Outcomes for PICOT # 1: Primary and secondary outcomes related to TB treatment
  Comment: ‘Death’ has been taken as Primary TB treatment outcome. Looking to the objective no.1, outcome would be the improvement in TB treatment success. ‘Death’ is a part of the unsuccessful TB treatment outcome, along with ‘loss to follow up’, ‘treatment failure’ etc. Therefore, for primary TB treatment outcome(s), author can either take only one outcome ‘TB treatment success rate’ or two outcomes ‘TB treatment success’ and ‘TB treatment unsuccess’. The outcome ‘Death’ can be shifted to secondary TB treatment outcomes.
**b.** Outcomes for PICOT # 2: Primary and secondary outcomes related to socioeconomic outcomes
  Comment: Outcomes are not prioritized as primary and secondary outcomes. It seems that authors have used main headings for primary outcomes, and sub-headings as secondary outcomes. However, prioritization needs to be mentioned clearly in the table, or as a footnote.
2.
**Search Strategy**

**a.** It would be better if authors mention about whether they will use any filters or not? If yes, what type of filters? Also, there is no mention about qualification of searcher.
**b**. Search strategy keywords (Table 2)
  1. Tuberculosis (“TB treatment terms”) - add ‘people with TB’ as keyword, the widely used phrase for TB patients.
  2. Social protection (“intervention terms”) - add ‘direct benefit transfer’, ‘DBT’ as keywords. In India, one of the high burden country for TB, monthly cash given to TB patients as direct benefit transfer in their bank accounts as social protection intervention. The phrase ‘Direct benefit transfer’ and ‘DBT’ are so prevalent that many a times these words are used instead of ‘cash transfer’ in the literature. If ‘Direct benefit transfer’ and ‘DBT’ will                not be used as keywords, there are chances to miss important relevant articles.
  3. Treatment (“outcome terms”) – ‘Treatment failure’ is taken as one of the outcomes for PICOT #1, but not used in search strategy. ‘Treatment failure’ should be added as keyword.
3.
**Data items**

**a**. Definitions of listed variables are not available. If there is a constraint of word limits, authors may add supplementary file.
**b**. There is no statement on piloting forms, and any pre-planned data assumptions and simplifications.
4. **Risk of bias in individual studies**
**a.** No mention of experience of assessors- training, piloting, previous risk of bias assessment experience.
**b.** Authors need to describe ‘how risk of bias assessment will be incorporated into data synthesis (any plans to do subgroup or sensitivity analysis)’ and ‘their potential influence on findings of the review’.
5.
** Data synthesis**

**a.** Authors may write about threshold value of I ^2^, above which they will not pool results.
**b. **Authors can add ‘duration of social protection intervention after launch in XYZ country, at the time of study’ as explanatory variable. Because, during initial months or years of implementation of social protection intervention, there may be teething problems (especially in LMICs), which may affect the intervention effect.
**c. **Is there any plan for qualitative (narrative) synthesis? If yes, how? This needs to be stated.

Is the study design appropriate for the research question?

Yes

Is the rationale for, and objectives of, the study clearly described?

Yes

Are sufficient details of the methods provided to allow replication by others?

Yes

Are the datasets clearly presented in a useable and accessible format?

Partly

Reviewer Expertise:

Professor in medical school with expertise in research and epidemiology

I confirm that I have read this submission and believe that I have an appropriate level of expertise to confirm that it is of an acceptable scientific standard, however I have significant reservations, as outlined above.

# Reviewer report: Reviewer response for version 1

**DOI:** 10.21956/wellcomeopenres.20853.r63778

**Published:** 2023-09-07

**Authors:** Fernando Rubinstein

**Affiliations:** 1 Institute for Clinical Effectiveness and Health Policy (IECS), Buenos Aires,, Argentina

This is an interesting systematic review and eventual meta-analysis to update the impact of different social support strategies for TB patients (and their families) on treatment outcomes, both in terms of clinical and socioeconomic status.

 The present protocol describes very clearly a step-by-step standard meta-analysis plan and addresses potential limitations regarding the heterogeneity of the study designs, different interventions under the definition of social protection and the difficulties of reporting a pooled estimate of effect. The authors acknowledge the fact that only including studies published in English may limit the analysis and bias the results.

 The authors present detailed information of the search keywords and strategies, allowing the readers to reproduce the searches and update them when necessary.

 They plan to report the results stratified by outcome and, if the numbers allow, by type or group of interventions, although this seems to be more difficult to achieve given the diversity of definitions of social protection interventions and the implementation strategies.  Also, the heterogeneity of study designs and the different TB status of the participants in the studies may make difficult the comparison of results of similar interventions, but the authors state that if the heterogeneity is important (say, I2 > 50%) results will not be pooled.

 The authors plan to run a meta regression to evaluate the weight that predefined variables may have on the variability of the outcome. This is an interesting addition to the protocol since the majority of the meta-analyses report a pooled average measure of effect, not paying enough attention to explore the underlying heterogeneity of the results or the methods used in the different individual studies included. The meta-regression can help to explain residual heterogeneity considering study-level variables in the analysis, which could be further explained by differences in characteristics of the studies (methodological diversity) or study populations (clinical diversity).

 Finally, as noted by the authors, the results of this updated and comprehensive study should serve as a strong basis for stakeholders, policy makers and National TB Programs to make informed decisions about which social protection interventions should be incorporated, implemented and scaled-up to contribute to break the cycle of poverty and disease.

Is the study design appropriate for the research question?

Yes

Is the rationale for, and objectives of, the study clearly described?

Yes

Are sufficient details of the methods provided to allow replication by others?

Yes

Are the datasets clearly presented in a useable and accessible format?

Not applicable

Reviewer Expertise:

Epidemiology, TB, Research methods

I confirm that I have read this submission and believe that I have an appropriate level of expertise to confirm that it is of an acceptable scientific standard.
